# Ecological and Structural Drivers of Song Amplitude Variation in Passerines

**DOI:** 10.1002/ece3.74693

**Published:** 2026-11-10

**Authors:** K. Deoniziak, W. Mierzejewska, M. Nartowicz, D. Winiarska

**Affiliations:** ^1^ Division of Biodiversity and Behavioural Ecology, Faculty of Biology University of Bialystok Białystok Poland; ^2^ The Włodzimierz Chętnicki Biological Science Club, Faculty of Biology University of Bialystok Białystok Poland

**Keywords:** acoustic communication, bioacoustics, birds, signal transmission, song structure, vocal plasticity

## Abstract

Song amplitude is an important but comparatively understudied dimension of avian acoustic communication, influencing signal transmission, detectability and social interactions. We investigated ecological and structural drivers of song amplitude variation in six passerine species using 3265 songs recorded from 120 territorial males. Body size was associated with cross‐species variation in amplitude only when the largest and loudest species, the Great Reed Warbler, was included. Across species, song amplitude declined with increasing temperature, relative humidity and time after sunrise, whereas ambient noise showed no evidence of a generalised Lombard effect. Song rate, song length and peak frequency were consistent predictors of amplitude, with peak frequency showing the strongest association. Within‐species analyses revealed substantial heterogeneity in environmental effects, whereas relationships between amplitude and song structure were more consistent across species. Individual identity explained considerable variation in amplitude, indicating pronounced within‐species variability in vocal output. Overall, our results suggest that song amplitude is a flexible component of vocal performance shaped primarily by song structure and signalling context rather than by body size alone. These findings emphasise the importance of considering both behavioural and ecological sources of variation when interpreting patterns of acoustic signalling in birds.

## Graphical Abstract

Using calibrated measurements of 3265 songs from 120 males of six passerine species, we examined how song amplitude varies with body size, environmental conditions and song structure, both across and within species. Amplitude was most consistently associated with song structure and time of day, whereas weather effects were largely species‐specific and body size was associated with amplitude only when the largest species, the Great Reed Warbler, was included. These findings indicate that song amplitude is a flexible component of avian acoustic communication shaped by ecological and behavioural factors rather than a fixed species trait.

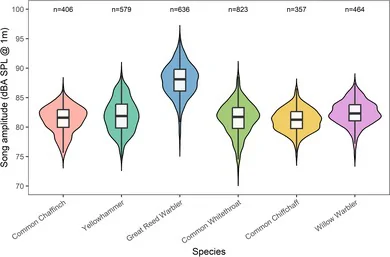

## 1 Introduction

In birds, vocalisations play central roles in territory defence, mate attraction and social coordination, making the factors that influence signal transmission and reception important targets for ecological and evolutionary research (Catchpole and Slater 2008). Among the properties that determine communication effectiveness, vocal amplitude reflects signal loudness and influences how far it can travel while remaining detectable to receivers (Wiley and Richards 1978; Bradbury and Vehrencamp 2011). Although birdsong has been studied extensively, song amplitude remains comparatively understudied relative to other acoustic traits, such as spectral and temporal features (e.g., Deoniziak and Osiejuk 2021; Cardoso et al. 2025). Recent comparative work demonstrates that it is highly plastic within and among species (Brumm et al. 2025), suggesting that vocal output should be considered a dynamic component of signal design rather than a fixed species characteristic. Despite growing evidence for such variability (e.g., Goto et al. 2025; de Framond et al. 2025), the mechanisms underlying it remain incompletely understood. In particular, the relative importance of biological and environmental influences remains unclear, as does the extent to which methodological decisions influence measurements of song amplitude.

Several mechanisms have been proposed to explain variation in song amplitude. Morphological traits can constrain the physical limits of vocal production (Zollinger et al. 2011), leading to expectations that larger species should produce louder vocalisations. However, evidence for such relationships remains mixed: positive associations have been reported across passerines, based on perceived loudness scores (Cardoso 2010), and across a broad range of bird families (Menezes and Podos 2026), but not in other comparisons across species (Brumm et al. 2025) or within species (Brumm 2009). Song amplitude has also been associated with acoustic parameters such as peak frequency, song duration and other measures of vocal performance (Cardoso 2010; Nemeth et al. 2013; Zollinger et al. 2012; Menezes and Podos 2025), indicating that amplitude may be integrated with other components of song structure.

Amplitude can also respond to environmental and social conditions. Environmental noise, atmospheric conditions and signalling context can influence both the production and transmission of acoustic signals (Brumm and Naguib 2009; Zollinger and Brumm 2015). So far, song amplitude has been linked to sexual and territorial signalling, with females of some species preferring louder songs (Searcy 1996; Ritschard et al. 2010), and males adjusting vocal output in territorial interactions (Brumm and Todt 2002; Brumm and Ritschard 2011; Acorn et al. 2026). Song amplitude may also increase in response to elevated background noise through the Lombard effect, a widespread behavioural adjustment that can improve signal detectability (Brumm and Zollinger 2011; Kunc et al. 2022). Understanding variation in amplitude is therefore increasingly important as anthropogenic noise reshapes acoustic environments, alters the conditions under which animals communicate, and influences the effectiveness of acoustic signalling in natural populations (Slabbekoorn and Ripmeester 2008; Duquette et al. 2021; Engel et al. 2024). Overall, no single factor appears sufficient to explain the observed variation, and amplitude plasticity appears to depend on an interaction between morphology, vocal production mechanisms, social context and environmental conditions (Brumm 2009; Brumm and Naguib 2009; Zollinger and Brumm 2015; Brumm et al. 2025).

Interpreting variation in song amplitude requires not only an understanding of its biological determinants, but also robust and consistent approaches to its measurement. Physically, amplitude is typically expressed as sound pressure level (SPL) at the point of emission, but the SPL received by a microphone, or by a conspecific, is shaped by multiple geometric and environmental factors, including distance, orientation, habitat structure, atmospheric conditions and sound degradation during transmission (Wiley and Richards 1978; Embleton 1996; Trujillo‐Torres et al. 2022; De Araújo et al. 2026). As a result, estimates of song amplitude depend on calibration procedures, recording geometry and analytical decisions.

Among these, the choice of weighting filter has received relatively little attention despite its potential influence on measured SPL values. Accurate estimation of song amplitude requires calibrated equipment and explicit documentation of recording conditions (Dabelsteen 1981; Zollinger et al. 2012; Zollinger and Brumm 2015; Brumm et al. 2025), but differences among weighting scales additionally affect the resulting amplitude estimates. A‐, C‐ and Z‐weighting apply different frequency‐dependent transformations to sound pressure levels: A‐weighting strongly attenuates low frequencies to approximate human auditory sensitivity at moderate sound levels, C‐weighting is considerably flatter, and Z‐weighting provides an unweighted response across the measured frequency range (Krug 1998). Because bird songs differ markedly in spectral composition, weighting choice may influence measured SPL in biologically important ways (Zollinger et al. 2012). Yet systematic comparisons of weighting scales for natural bird song remain scarce, limiting cross‐study comparability (Iglesias‐Merchan et al. 2024).

Here, we investigate the ecological and structural drivers of song amplitude in six passerine species. First, we examine cross‐species variation using three models to test whether body size, environmental and contextual variables, and song structure predict amplitude. Second, we analyse within‐species variation in amplitude using analogous models that separate contextual predictors from song‐structure predictors. Finally, we report amplitudes measured using A‐, C‐ and Z‐weighting to document the magnitude of differences among these scales for natural song.

## 2 Methods

### 2.1 Data Collection

Field recordings and SPL measurements were conducted in 2024 and 2025 in forest, agricultural and wetland habitats around Białystok, north‐eastern Poland (53.132720° N, 23.139522° E). We sampled six common passerine species, obtaining data from 20 males per species: Common Chaffinch *Fringilla coelebs* (406 analysed songs), Yellowhammer *Emberiza citrinella* (579 analysed songs), Great Reed Warbler *Acrocephalus arundinaceus* (636 analysed songs), Common Whitethroat *Curruca communis* (823 analysed songs), Common Chiffchaff *Phylloscopus collybita* (357 analysed songs), and Willow Warbler *Phylloscopus trochilus* (464 analysed songs). We recorded singing territorial individuals during territory establishment, which we assumed to be males; female song cannot be fully excluded, particularly in sexually monomorphic species (Sierro et al. 2022). All measurements were taken during the first 2 weeks after spring arrival from the wintering grounds to ensure that individuals were sampled at comparable stages of the breeding cycle. To minimise the risk of resampling the same individuals across years, no recordings were made in the same localities during the second field season. Fieldwork was restricted to days without precipitation and with minimal wind to minimise weather‐related variation in amplitude measurements.

Our procedures broadly followed established SPL measurement protocols for birdsong (Dabelsteen 1981; Brumm 2004; Brumm et al. 2025), while incorporating modifications suited to our study design and the comparison of multiple weighting scales. SPL measurements were obtained using a PCE‐430 Class 1 sound level meter (PCE Instruments, Germany), calibrated at the start of each field day using a PCE‐SC43 acoustic calibrator (94 dB SPL at 1 kHz). For each song, the instrument automatically recorded amplitude using A‐, C‐ and Z‐weighting with FAST exponential time weighting (0.125 s). Song amplitude was quantified as the maximum SPL (Lmax) reached during each complete song. Song duration varied among species (Table 1). No noise‐reduction, amplitude normalisation or dynamic‐range compression procedures were applied prior to acoustic analyses. SPL values were converted to source levels at 1 m using the inverse‐square law for geometric spreading (Brumm and Ritschard 2011). Because excess attenuation was not accounted for, source levels of the most distant songs may be slightly underestimated. To compare amplitudes across weighting functions within species, we used Kruskal–Wallis tests followed by Dunn's post hoc comparisons with Holm correction. Ambient noise was quantified as the maximum A‐weighted SPL measured during a 1‐s interval immediately following each recorded song. Summary statistics for ambient noise recorded during song measurements are provided in Table S1 for each species.

**TABLE 1:** Sample sizes and summary statistics for the six study species. Acoustic parameters were extracted in Avisoft SASLab Pro. SPL values are given in dB re 20 μPa at 1 m. Values are means ± SD, with ranges in parentheses.

Species	Scientific name	*N* songs	Song rate (per min)	Song length (s)	Peak frequency (Hz)	Min frequency (Hz)	Song amplitude dB(A)	Song amplitude dB(C)	Song amplitude dB(Z)
Common Chaffinch	*Fringilla coelebs*	406	8.2 ± 2.3 (4.7–14.1)	2.3 ± 0.4 (1.3–3.5)	3888 ± 187 (3090–4490)	1930 ± 189 (1370–2620)	81.3 ± 2.3 (74.8–86.6)	80.1 ± 2.1 (74.2–89.5)	81.6 ± 2.3 (76.4–95.4)
Yellowhammer	*Emberiza citrinella*	579	6.5 ± 1.1 (4.4–10.3)	2.3 ± 0.4 (1.4–3.3)	5303 ± 366 (4550–6490)	3894 ± 522 (2620–5160)	81.9 ± 2.8 (74.7–88.9)	81.2 ± 2.5 (75.2–88.0)	85.2 ± 4.7 (78.0–105.7)
Common Chiffchaff	*Phylloscopus collybita*	357	7.3 ± 1.1 (4.7–10.2)	4.6 ± 2.0 (2.0–15.9)	4279 ± 168 (3830–5010)	3156 ± 154 (2710–3570)	81.2 ± 1.9 (75.0–86.6)	80.2 ± 2.0 (73.8–94.3)	82.7 ± 2.9 (75.6–99.4)
Willow Warbler	*Phylloscopus trochilus*	464	7.3 ± 1.2 (5.6–10.5)	3.1 ± 0.5 (1.6–5.3)	4238 ± 247 (3340–5440)	2269 ± 222 (2020–3270)	82.4 ± 2.1 (74.9–87.4)	81.1 ± 2.0 (73.9–89.6)	83.4 ± 2.9 (77.1–110.8)
Common Whitethroat	*Curruca communis*	823	12.2 ± 2.4 (6.8–20.2)	1.5 ± 0.5 (0.5–4.9)	3826 ± 213 (3220–4680)	2121 ± 318 (1110–3180)	81.5 ± 2.8 (71.8–89.3)	80.5 ± 3.0 (71.0–94.5)	83.8 ± 6.2 (72.7–115.9)
Great Reed Warbler	*Acrocephalus arundinaceus*	636	8.1 ± 1.0 (5.9–13.5)	4.3 ± 1.4 (1.8–12.8)	2851 ± 235 (2020–3400)	1222 ± 377 (600–2360)	87.9 ± 2.8 (77.0–95.2)	86.5 ± 3.0 (75.7–96.8)	88.9 ± 5.4 (76.5–110.9)

Distances to singing males were measured for every song using two calibrated Vortex Viper HD3000 laser rangefinders (Vortex Optics, USA). Recording distances ranged from 5.1 to 25.2 m (mean ± SD = 15.5 ± 3.8 m). Because received amplitude is influenced by the directional radiation patterns of bird vocalisations, only songs produced while the bird was facing the sound level meter were retained for analysis (Larsen and Dabelsteen 1990; Patricelli et al. 2007; Zollinger et al. 2012). During each recording, both the sound level meter and the recording microphone were oriented directly towards the singing bird and positioned approximately 1.8 m above ground level to ensure a consistent measurement geometry. High‐quality audio recordings (WAV format, 44.1 kHz sampling rate, 24‐bit resolution) were collected simultaneously using a Sound Devices MixPre‐3 II recorder (Sound Devices, USA) and a Sennheiser MKH8070 shotgun microphone (Sennheiser, Germany) fitted with a Rycote WS 8J windshield (Rycote Microphone Windshields Ltd., UK). Behavioural notes, including distance, orientation, perch height and movements, were recorded in real time via a Røde Lavalier microphone (Røde Microphones, Australia) connected to the recorder's second channel. Temperature and relative humidity were monitored with DS1923 iButton loggers (Maxim Integrated, USA) positioned 1 m above ground and programmed to record at 60‐s intervals. For each focal song, we extracted the temperature and humidity values closest in time to the recording.

Subsequent ecological analyses were conducted using A‐weighted measurements because this weighting scale is the most widely reported metric in studies of avian song amplitude and facilitates comparison with previous work (e.g., Brumm 2004; Brumm et al. 2025), and because the A‐filter, which is approximately flat between c. 1 and 8 kHz and attenuates lower and higher frequencies, broadly approximates avian hearing sensitivity (Brumm 2004; Dooling et al. 2000).

### 2.2 Across‐Species Analysis of Song Amplitude

We quantified interspecific variation in song amplitude using Bayesian linear mixed models (LMM) with Gaussian errors, fitted with the stan_glmer function in the rstanarm package (Goodrich et al. 2025) in R (R Core Team 2025). Song amplitude (dB(A) SPL re 20 μPa at 1 m) was the response variable. All across‐species models included species identity and male identity as random intercepts, and recordist identity as a fixed effect. Continuous predictors were centred and scaled prior to analysis as described below, except hour after sunrise, which was entered in hours. Posterior distributions were estimated using four Markov chains of 10,000 iterations each, including 5000 warm‐up iterations, under default weakly informative priors. For the across‐species models, the target acceptance rate (adapt_delta) was set to 0.995 and the maximum tree depth to 15. Convergence and model fit were assessed using trace plots, R̂, effective sample sizes and posterior predictive checks (bayesplot; Gabry and Mahr 2025). We report posterior means and 95% credible intervals, and consider an effect supported when its 95% credible interval excludes zero.

In Model 1, we tested whether body size predicts variation in amplitude using a species‐level morphological index derived from principal component analysis (PCA) of AVONET morphological data (Tobias et al. 2022). We extracted species means for body mass, tarsus length, wing length and culmen length, and reduced these variables to a single composite index using PCA. The first component (PC1) explained 75% of the total variance and was used as an index of overall body size (Table S2). This index was centred and scaled by one standard deviation. Because the Great Reed Warbler is markedly larger than the other species, we repeated Model 1 after excluding it, re‐standardising the body‐size index on the reduced data set. This reduced model produced 175 divergent transitions (0.9% of post‐warm‐up draws; 5 in the full model), reflecting a between‐species variance close to zero, and its estimates should be interpreted with corresponding caution.

In Model 2, we tested environmental and contextual predictors of SPL: temperature, relative humidity, ambient noise, hour after sunrise and the presence of a singing conspecific. Temperature (°C) was centred at 12 and scaled by 5; humidity (%) was centred at 55 and scaled by 10; and ambient noise (dB(A)) was centred at 40 and scaled by 6 (Brumm et al. 2025). Hour after sunrise was not rescaled. Conspecific presence was coded as a binary variable (0 = absent, 1 = present).

In Model 3, we examined whether song rate, song length, peak frequency and minimum frequency predicted amplitude. Acoustic parameters were extracted in Avisoft SASLab Pro v.5.3.2 (Avisoft Bioacoustics, Germany) using FFT size = 1024, a Hamming window, 75% overlap, and a high‐pass filter at 500 Hz. An amplitude threshold of −12 dB relative to the spectral peak was used to delimit signal from background noise. Recordings affected by overlap with biotic or anthropogenic noise were excluded after visual inspection. All acoustic predictors were centred and scaled by one standard deviation across the full data set.

### 2.3 Within‐Species Analysis

To quantify individual‐level amplitude variation, we fitted two Bayesian linear mixed models per species: one with contextual predictors (temperature, relative humidity and hour after sunrise; Model 1) and one with acoustic predictors (song rate, song length and peak frequency; Model 2). Predictor sets were selected based on patterns identified in the across‐species analyses. Predictors were scaled exactly as in the across‐species models: temperature and humidity with the fixed centring and scaling values given above, hour after sunrise in hours, and acoustic predictors standardised using the mean and standard deviation of the full data set, so that coefficients are expressed in the same units in all species. Male identity was included as a random intercept. Recordist identity was not included, because it had no supported effect in any across‐species model and one species was recorded by a single recordist. Random slopes were not fitted, to maintain model stability given the smaller sample sizes within species. Models were fitted using stan_glmer with four chains, 10,000 iterations per chain and 5000 warm‐up iterations, under default priors and sampler settings. Posterior summaries are reported as means and 95% credible intervals. As in the across‐species analyses, convergence was evaluated using effective sample sizes and inspection of trace plots.

## 3 Results

### 3.1 General Results

We analysed 3265 songs recorded from 120 territorial males belonging to six species (20 males per species). The Great Reed Warbler had the highest mean amplitude of 87.9 dB(A), whereas the remaining species clustered within a narrow range of approximately 81–82 dB(A) (Table 1, Figure 1). Song amplitude at 1 m differed significantly among weighting scales (A, C and Z) in all species (Kruskal–Wallis tests; all *p* < 0.001): Common Chaffinch *χ* ^2^ = 98.9; Yellowhammer *χ* ^2^ = 320.0; Common Chiffchaff *χ* ^2^ = 204.0; Willow Warbler *χ* ^2^ = 207.0; Common Whitethroat *χ* ^2^ = 197.0; and Great Reed Warbler *χ* ^2^ = 88.6 (d.f. = 2 in all cases). Dunn post hoc tests with Holm correction showed that A‐ and C‐weighted values, and C‐ and Z‐weighted values, differed in all six species, whereas A‐ and Z‐weighted values differed in four species but not in the Common Chaffinch or Great Reed Warbler (Table S3). Across species, Z‐weighted measurements consistently yielded higher amplitude estimates than A‐weighted values, whereas C‐weighted values were consistently the lowest, c. 0.7–1.4 dB below A‐weighted values (Table 1). These results indicate that song amplitude estimates depend systematically on the weighting scale used.

**FIGURE 1:**
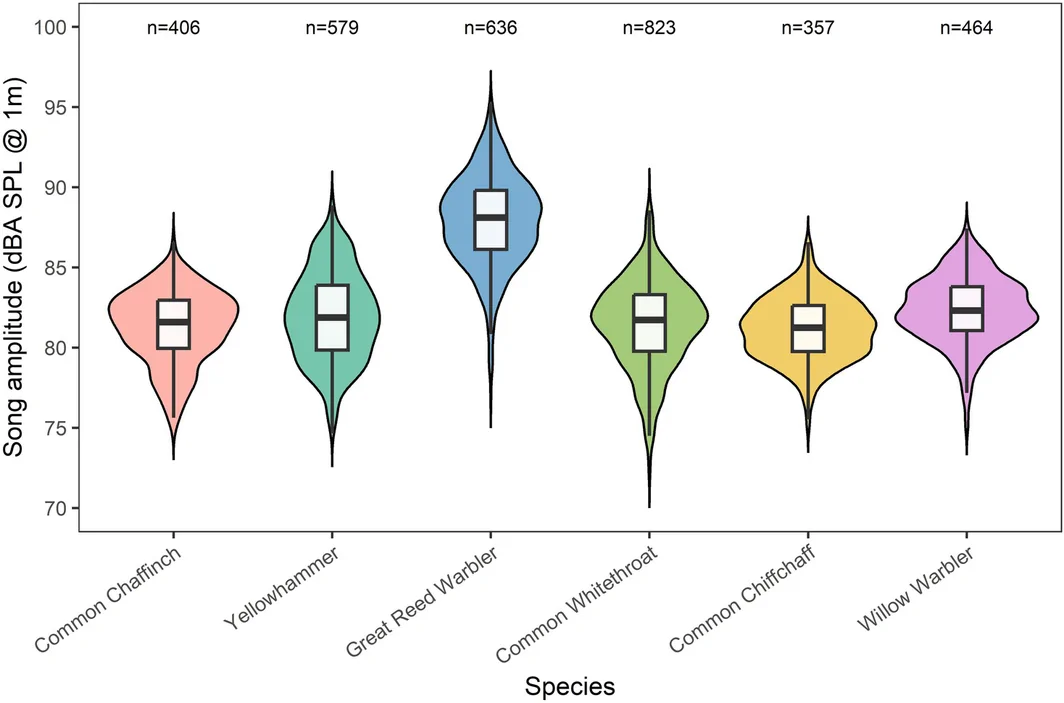
Distribution of A‐weighted song amplitude (dB(A) re 20 μPa at 1 m) across species. Violins show kernel density estimates and boxplots indicate medians and interquartile ranges. Sample sizes for analysed songs are given above each violin.

### 3.2 Across‐Species Analysis

#### 3.2.1 Body Size Effects on Amplitude (Model 1)

Across the six passerine species, song amplitude increased with body size (Table 2). The LMM revealed a positive relationship between standardised body size and song amplitude, indicating that larger species produced louder songs on average. Recordist identity had no measurable effect on amplitude, suggesting little observer‐related bias. Random‐effect estimates indicated substantial variation among species (SD = 1.91) and slightly greater variation among individuals within species (SD = 2.07). However, when Great Reed Warbler was excluded from the model, the effect of body size on song amplitude decreased and was no longer supported (Table S4).

**TABLE 2:** Posterior estimates from three Bayesian linear mixed models testing predictors of song amplitude across six passerine species. Song amplitude (dB(A) re 20 μPa at 1 m) was the response variable. Model 1 tested the effect of body size while controlling for recordist identity, with species and male identity fitted as random intercepts. Model 2 examined environmental and contextual predictors: temperature, relative humidity, ambient noise, presence of a singing conspecific, hour after sunrise and recordist identity. Model 3 examined song‐structural predictors: song rate, song length, peak frequency, minimum frequency and recordist identity. All continuous predictors were centred and scaled as described in the main text. Values are posterior means with 95% credible intervals. Effects whose credible intervals do not overlap zero are shown in bold.

Model	Predictor	Posterior mean (95% CI)
Model 1—Body size	**(Intercept)**	**82.80 (81.16 to 84.44)**
	**Body size (PC1)**	**1.83 (0.25 to 3.42)**
	Recordist	−0.33 (−1.29 to 0.58)
Model 2—Contextual and environmental predictors	**(Intercept)**	**84.11 (81.72 to 86.61)**
	**Hour after sunrise**	**−0.52 (−0.90 to −0.16)**
	**Temperature**	**−0.46 (−0.76 to −0.16)**
	**Humidity**	**−0.25 (−0.47 to −0.04)**
	Ambient noise	−0.10 (−0.19 to 0.0006)
	Singing conspecifics	−0.22 (−0.68 to 0.23)
	Recordist	−0.52 (−1.44 to 0.45)
Model 3—Song characteristics	**(Intercept)**	**82.61 (79.93 to 85.18)**
	**Song rate**	**0.27 (0.04 to 0.49)**
	**Song length**	**0.12 (0.01 to 0.22)**
	**Peak frequency**	**0.69 (0.43 to 0.94)**
	Minimum frequency	0.15 (−0.09 to 0.38)
	Recordist	−0.40 (−1.33 to 0.52)

#### 3.2.2 Contextual and Environmental Effects on Amplitude (Model 2)

Contextual and environmental conditions were associated with variation in song amplitude (Table 2). Temperature had a negative effect on SPL, with birds singing more quietly under warmer conditions. Relative humidity also showed a negative association with amplitude. Ambient noise showed a weak negative trend, but the credible interval overlapped zero, providing no evidence for a Lombard effect at the across‐species level. The presence of another singing conspecific had no detectable effect. In contrast, hour after sunrise was negatively related to amplitude, with louder songs produced earlier in the morning. Recordist identity again had no measurable effect. Random‐effect estimates indicated considerable variation at both the species and individual levels.

#### 3.2.3 Song‐Structure Effects on Amplitude (Model 3)

Three structural features of song were associated with variation in amplitude (Table 2). Song rate was positively related to SPL, with faster singers producing louder songs. Song length also showed a positive effect, indicating slightly higher amplitudes in longer songs. Peak frequency was the strongest predictor, with higher‐frequency songs sung at higher amplitudes. A one standard deviation increase in peak frequency corresponded to an increase of approximately 0.7 dB in song amplitude. Minimum frequency showed no clear effect. Recordist identity again had no measurable influence on amplitude. Random‐effect estimates indicated substantial variation among both species and individuals, with species accounting for the larger share of variation.

### 3.3 Within‐Species Analysis

#### 3.3.1 Contextual Predictors (Model 1)

The effects of abiotic and temporal predictors on song amplitude varied markedly among species (Table 3). Great Reed Warblers showed the strongest contextual effects. Song amplitude declined with hour after sunrise, temperature and relative humidity, indicating that males produced their loudest songs early in the morning and under cooler, drier conditions. Relative humidity was associated with song amplitude in two additional species, but in opposite directions. In Yellowhammers, humidity had a negative effect, whereas in Chaffinches it had a positive effect. Willow Warblers showed a consistent negative effect of temperature, whereas humidity and hour after sunrise had no clear effects. By contrast, Whitethroats and Chiffchaffs showed no credible effects of any contextual predictor, indicating that song amplitude showed no detectable association with the measured environmental variables in these species.

**TABLE 3:** Posterior estimates from within‐species Bayesian linear mixed models testing contextual and song‐structural predictors of song amplitude in six passerine species. Models were fitted separately for each species, with male identity included as a random intercept. Shown are posterior means and 95% credible intervals for fixed effects from the contextual models (temperature, relative humidity and hour after sunrise) and the song‐structure models (song rate, song length and peak frequency). Effects whose credible intervals do not overlap zero are shown in bold.

Model	Species	Predictor	Posterior mean (95% CI)
Contextual	Common Chaffinch	**(Intercept)**	**81.61 (78.79 to 84.67)**
		Temperature	0.23 (−0.76 to 1.20)
		**Humidity**	**0.85 (0.20 to 1.50)**
		Hour after sunrise	−0.65 (−1.63 to 0.22)
	Yellowhammer	**(Intercept)**	**83.58 (80.08 to 86.86)**
		Temperature	−0.31 (−2.07 to 1.33)
		**Humidity**	**−1.62 (−2.71 to −0.61)**
		Hour after sunrise	0.73 (−0.86 to 2.60)
	Great Reed Warbler	**(Intercept)**	**93.70 (90.72 to 97.12)**
		**Temperature**	**−1.04 (−1.73 to −0.35)**
		**Humidity**	**−0.92 (−1.68 to −0.17)**
		**Hour after sunrise**	**−2.48 (−3.78 to −1.29)**
	Common Whitethroat	**(Intercept)**	**81.44 (79.02 to 83.89)**
		Temperature	0.33 (−0.50 to 1.18)
		Humidity	−0.01 (−0.62 to 0.59)
		Hour after sunrise	−0.28 (−1.24 to 0.68)
	Common Chiffchaff	**(Intercept)**	**82.15 (80.11 to 83.98)**
		Temperature	−0.30 (−1.37 to 0.93)
		Humidity	−0.29 (−0.83 to 0.31)
		Hour after sunrise	−0.23 (−1.12 to 0.66)
	Willow Warbler	**(Intercept)**	**82.48 (81.17 to 84.00)**
		**Temperature**	**−1.11 (−1.59 to −0.63)**
		Humidity	−0.17 (−0.41 to 0.08)
		Hour after sunrise	−0.04 (−0.68 to 0.48)
Song characteristics	Common Chaffinch	**(Intercept)**	**81.41 (80.47 to 82.35)**
		Song rate	0.36 (−0.09 to 0.81)
		Song length	−0.41 (−1.09 to 0.24)
		**Peak frequency**	**1.12 (0.49 to 1.75)**
	Yellowhammer	**(Intercept)**	**79.16 (77.60 to 80.71)**
		**Song rate**	**−2.33 (−3.55 to −1.05)**
		Song length	0.48 (−0.33 to 1.29)
		**Peak frequency**	**0.62 (0.14 to 1.10)**
	Great Reed Warbler	**(Intercept)**	**87.79 (86.31 to 89.25)**
		**Song rate**	**1.10 (0.30 to 1.90)**
		**Song length**	**0.33 (0.15 to 0.51)**
		Peak frequency	0.27 (−0.43 to 0.99)
Song characteristics	Common Whitethroat	**(Intercept)**	**80.06 (78.71 to 81.38)**
		**Song rate**	**0.59 (0.27 to 0.91)**
		Song length	−0.34 (−0.80 to 0.10)
		**Peak frequency**	**0.76 (0.24 to 1.28)**
	Common Chiffchaff	**(Intercept)**	**80.47 (79.71 to 81.25)**
		**Song rate**	**−1.19 (−1.79 to −0.59)**
		Song length	−0.08 (−0.21 to 0.04)
		Peak frequency	0.45 (−0.49 to 1.39)
	Willow Warbler	**(Intercept)**	**81.85 (81.05 to 82.63)**
		Song rate	−0.15 (−0.82 to 0.54)
		**Song length**	**0.67 (0.11 to 1.22)**
		**Peak frequency**	**0.99 (0.43 to 1.54)**

#### 3.3.2 Song‐Structure Predictors (Model 2)

Song characteristics were more consistently associated with amplitude than contextual variables, although the direction and strength of effects differed among species (Table 3). In Great Reed Warblers, both song rate and song length positively predicted amplitude, indicating that males produced longer and faster songs at higher amplitude. Yellowhammers showed a strong negative relationship between song rate and amplitude, but a positive effect of peak frequency. In Chaffinches, amplitude increased with peak frequency, while song rate and song length showed no clear effects. Chiffchaffs also showed a negative effect of song rate, with no detectable effects of song length or peak frequency. In Willow Warblers, song amplitude was positively associated with both song length and peak frequency. Finally, Whitethroats showed positive effects of song rate and peak frequency, whereas song length did not clearly predict amplitude.

## 4 Discussion

Using calibrated field measurements and a hierarchical analytical framework, we investigated variation in song amplitude across and within six passerine species. Our results show that song amplitude is shaped primarily by song structure and temporal context, whereas environmental effects appeared species‐specific and body size was associated with interspecific variation only when the Great Reed Warbler was included. As expected from the filter characteristics, amplitude estimates also differed among weighting scales. Taken together, these findings support the view that song amplitude is a flexible component of acoustic signal design that varies with signalling context, song structure and environmental conditions, rather than representing a fixed species‐level trait.

Individual identity explained a substantial proportion of variance in all models and, in the body‐size analysis, its contribution was slightly greater than that of species identity (SDindividual = 2.07 vs. SDspecies = 1.91). This pronounced within‐species variation suggests that song amplitude is strongly modulated at the individual level, likely reflecting differences in motivation, social context and signalling role rather than anatomical constraints alone. Comparable inter‐individual variation in vocal amplitude has been documented repeatedly under natural conditions (e.g., Dabelsteen 1981; Brumm and Todt 2002; Liu et al. 2023; Brumm et al. 2025; Acorn et al. 2026).

### 4.1 Interspecific Variation and the Role of Body Size

Across species, song amplitude increased with body size, but this relationship was driven largely by the inclusion of the Great Reed Warbler, whose songs were consistently louder than those of the other species studied. When this species was excluded, body size no longer predicted amplitude, indicating that simple allometric scaling explained little of the variation in song amplitude among the species studied here. This result aligns with comparative analyses across diverse taxa showing that most acoustic signals exhibit shallow static allometries, low coefficients of variation and limited dispersion around the allometric function, and therefore rarely function as reliable indicators of body size at the individual or species level (Rodríguez et al. 2015). Such shallow allometries may arise because behavioural signals can be flexibly modulated across contexts, even when produced by anatomical structures that might otherwise be expected to constrain signal expression (Rodríguez et al. 2015; West‐Eberhard 2003). Brackenbury (1979), who measured maximum song SPL in 17 British songbirds, including five of the species studied here, likewise found that larger species produced more sound power overall, but could not establish a consistent relationship between body size and vocal performance. Notably, the Common Whitethroat was the quietest species in his sample (74 dB at 1 m), whereas in our data its amplitude was similar to that of the other small species, which may partly reflect differences in measurement methods, as levels were read from a needle meter and distances measured with string. Together with passerine‐focused analyses showing weak or inconsistent size–amplitude relationships (Brumm 2009; Brumm et al. 2025), this supports the view that amplitude is poorly suited as a general index of body size within restricted taxonomic groups.

At broader phylogenetic scales, however, body size may still impose upper limits on signal amplitude, particularly when signals are produced under relatively unconstrained conditions. Jurisevic and Sanderson (1998) reported positive relationships between body mass and SPL in avian distress calls across multiple orders. In a comparative analysis of 123 species from 39 families, Menezes and Podos (2026) likewise found that maximum amplitude increased with body size and, more robustly, with beak gape width, although the body‐size effect became uncertain when species represented by a single individual were excluded. If beak dimensions contribute to amplitude, they may also help explain the high amplitude of the Great Reed Warbler, which has by far the longest culmen of our study species (Table S2). Similarly, comparative work on Arctic‐breeding birds reported a positive, phylogenetically structured association between body mass and estimated source amplitude across genera (Ziegenhorn et al. 2026). While these broader‐scale patterns are informative, source‐level estimates in the latter study were based on visually estimated distances rather than laser range‐finding or equivalent calibrated methods, which may introduce additional uncertainty into amplitude calculations. As a result, the strength of the reported size–amplitude relationship should be interpreted with caution.

Importantly, these broader‐scale patterns do not contradict our findings. Instead, they suggest that size‐related constraints on amplitude may emerge across wide evolutionary distances or under optimal signalling conditions, but become much weaker within a limited passerine assemblage, where signal expression is strongly shaped by behavioural flexibility, context and environmental constraints. Experimental work in other taxa further supports this interpretation by demonstrating that environmental conditions can modulate size‐dependent signalling in ways that weaken or even reverse expected allometric patterns. For example, in túngara frogs, call amplitude reliably reflects male size only under favourable display‐site conditions, whereas environmental constraints can eliminate or invert size–amplitude relationships under suboptimal conditions (Smit et al. 2019). These findings reinforce the conclusion that body size alone provides an incomplete and context‐dependent explanation for variation in acoustic signal amplitude.

### 4.2 Contextual Modulation and Environmental Effects

At the across‐species level, ambient noise did not predict increased song amplitude, providing no evidence for a generalised Lombard effect under the conditions sampled. This contrasts with the strong across‐species Lombard effect reported by Brumm et al. (2025), as well as with laboratory studies and work in chronically noisy or urban habitats, where consistent noise‐dependent amplitude regulation has been documented (Cynx et al. 1998; Brumm and Todt 2002; Leonard and Horn 2005; Kunc et al. 2022). However, experimental work reviewed by Brumm and Zollinger (2011) shows that Lombard responses are not elicited by noise per se, but depend on its persistence, spectral overlap and masking efficacy relative to the signal. In both birds and mammals, amplitude regulation is strongest when background noise overlaps the vocal signal and remains intense enough to reduce effective signal‐to‐noise ratios (Sinnott et al. 1975; Manabe et al. 1998; Brumm and Todt 2002; Brumm and Zollinger 2011).

From an environmental‐acoustics perspective, the absence of a consistent Lombard effect in our study is therefore not surprising. In natural habitats, background noise is often transient and spatially heterogeneous, arising from wind, vegetation movement, insects and heterospecific vocalisations, and is unlikely to generate sustained masking over the time scales relevant for song production. Under such conditions, selection may favour alternative strategies for maintaining communication efficiency, including temporal avoidance of noise peaks, perch choice, signal directionality, or redundancy in signal structure, rather than systematic increases in amplitude (e.g., Brumm and Slater 2006; Gough et al. 2014). Consistent with this view, experimental studies show that animals may alter call timing, signal duration or repetition rate in noise even when amplitude remains unchanged (Potash 1972; Cynx et al. 1998; Brumm and Zollinger 2011). The absence of an across‐species Lombard effect in our data should therefore not be taken as evidence against amplitude plasticity, but rather as evidence that noise‐dependent amplitude regulation is conditional and context‐dependent.

Time after sunrise was negatively associated with song amplitude in the across‐species models, with birds singing louder earlier in the morning. By contrast, within‐species analyses revealed a credible temporal decline in amplitude only in the Great Reed Warbler, whereas the remaining species showed no clear within‐species effect of time after sunrise. This pattern suggests that the strong across‐species temporal signal reflects a general tendency for elevated vocal output during early morning hours, while species‐specific temporal modulation is more limited and context‐dependent. Importantly, early morning singing coincides not only with peak territorial and mating activity, but also with microclimatic conditions that are optimal for sound transmission. Classic modelling work by Henwood and Fabrick (1979) demonstrated that, from the perspective of atmospheric attenuation alone, early morning conditions can maximise broadcast area relative to later in the day, in some environments increasing effective coverage by an order of magnitude compared with midday conditions. Elevated song amplitude at dawn may therefore reflect an interaction between behavioural motivation and favourable transmission conditions, rather than a purely social or purely physical driver (Morton 1975; Catchpole and Slater 2008; Brumm and Naguib 2009).

Temperature and relative humidity showed negative associations with amplitude at the across‐species level, indicating lower SPL under warmer and more humid conditions. Physical acoustics predicts that atmospheric conditions influence sound transmission through changes in molecular absorption, sound speed, refraction and turbulence, with particularly strong and often non‐linear effects at mid to high frequencies (Richards and Wiley 1980; Embleton 1996). Experimental and modelling studies further show that increases in air temperature and humidity can alter attenuation and reverberation in ways that reduce signal stability and effective transmission range, especially under fluctuating outdoor conditions (Benedetto and Spagnolo 1983; Winkler‐Skalna 2025).

At the same time, weather effects in the field were tightly linked to time after sunrise, with temperature generally increasing and relative humidity declining as the morning progressed. This covariation complicates the separation of physical and behavioural processes, particularly because the rate of humidity loss differs among habitats. Open landscapes, such as those occupied by Yellowhammers and Great Reed Warblers, typically lose humidity rapidly after sunrise, whereas forest interiors retain moisture for longer. Common Chaffinches were recorded mainly in forest interiors, where humidity remained elevated later into the morning and where sound transmission is more strongly shaped by reverberation and scattering than by simple atmospheric attenuation (Marten and Marler 1977; Richards and Wiley 1980; Dabelsteen et al. 1993). This habitat‐specific microclimate may help explain why humidity showed a positive association with amplitude in Chaffinches but negative or neutral effects in open‐habitat species. In forest environments, higher humidity may coincide with early‐morning social peaks and relatively stable transmission conditions, whereas in open habitats declining humidity may simply track reduced signalling effort later in the day. Overall, the effects of time after sunrise, temperature and humidity on song amplitude appear tightly interwoven and mediated by habitat‐specific microclimates and signalling strategies, rather than reflecting uniform atmospheric constraints across species.

### 4.3 Song Structure as a Primary Determinant of Amplitude

Song‐structural variables were the most consistent predictors of amplitude across species. Higher song rates, longer songs, and especially higher peak frequencies were associated with increased SPL, indicating that amplitude covaries with temporal and spectral song features rather than being regulated as an independent channel. This pattern is consistent with the biomechanics of sound production: increasing vocal amplitude requires higher subsyringeal air‐sac pressure and respiratory muscle activity, which can simultaneously elevate the vibration rate of syringeal tissues and hence frequency unless actively compensated (Beckers et al. 2003; Goller and Cooper 2004; Zollinger et al. 2011). Empirical work further suggests that amplitude and frequency often covary in birdsong, both within repertoires and in response to noise, although the strength and direction of this relationship vary among species and song types (Nelson 2000; Nemeth and Brumm 2010; Zollinger et al. 2012). By contrast, a comparative study of more than 140 passerine species found that species with higher‐pitched songs were perceived as quieter (Cardoso 2010), although the author noted that this may partly reflect the lower detectability of high frequencies to human observers. Together, these findings indicate that amplitude–frequency relationships vary in direction (Menezes and Podos 2025) and may depend on the level of analysis and on how amplitude is quantified. Part of the positive association between song length and amplitude may, however, be methodological: with FAST time weighting (125 ms), longer elements fill more of the integration window, and longer songs offer more opportunities to reach a maximum level.

Functionally, this integration of amplitude, frequency and temporal structure is likely to enhance signal efficacy. Songs that combine high amplitude with rapid temporal patterning and elevated frequencies may be more detectable and more resistant to masking, yet they are also more vulnerable to degradation by reverberation and atmospheric turbulence, especially in structurally complex habitats such as forests (Wiley and Richards 1978; Richards and Wiley 1980; Naguib 2003). Species‐specific combinations of song amplitude and structure can therefore be interpreted as adaptive compromises that balance signal range, information content and resistance to degradation under local acoustic conditions (Morton 1975; Marten and Marler 1977).

Recent syntheses also suggest that the production of loud songs is unlikely to be strongly constrained by direct metabolic costs, even for substantial increases in amplitude, but may instead be limited by social and ecological costs such as heightened aggression from rivals or increased detectability by unintended receivers (Zollinger et al. 2011; Zollinger and Brumm 2015). This interpretation is consistent with our results and suggests that the associations between song structure and amplitude are best viewed as components of integrated vocal performance strategies shaped by communicative function and social context, rather than as simple energetic trade‐offs. Animals are not necessarily expected to maximise signal transmission distance. In many communication systems, receivers occur well within the maximum active space of a signal, and additional increases in amplitude may provide little benefit while increasing conspicuousness to rivals, predators or unintended receivers. Studies of soft song and short‐range signalling further demonstrate that low‐amplitude vocalisations can function adaptively in aggressive and social interactions rather than simply representing weaker versions of long‐range signals (Searcy and Beecher 2009; Loning et al. 2022). At the same time, the within‐species analyses revealed pronounced heterogeneity in structure–amplitude relationships, including positive, negative and null effects of song rate on amplitude. This cautions against universal interpretations of amplitude–structure trade‐offs and indicates that amplitude may serve different communicative roles across species and contexts, ranging from long‐range territorial advertisement to short‐range aggressive or courtship interactions (Searcy and Beecher 2009; Brumm and Naguib 2009; Zollinger and Brumm 2015).

### 4.4 Methodological Considerations in Song Amplitude Measurement

As expected from the filter characteristics, song amplitude estimates differed systematically across A‐, C‐ and Z‐weighting scales in all species. Although these filters were developed to approximate human hearing sensitivity, they impose distinct frequency‐dependent transformations on recorded signals. Because bird songs vary widely in spectral composition, weighting choice can substantially alter SPL estimates, particularly for broadband songs or those with pronounced low‐frequency components. As emphasised by bioacoustic theory and measurement studies, weighting filters can therefore introduce non‐biological variation into amplitude estimates that is unrelated to vocal output at the source (Krug 1998; Zollinger et al. 2012).

Work on sound transmission and detection further shows that frequency‐dependent attenuation, directionality and habitat structure translate directly into differences in effective detection range and signal salience, highlighting the need for biologically meaningful measurement standards (Marten and Marler 1977; Wiley and Richards 1978; Yip et al. 2017; Winiarska et al. 2024). Recent syntheses on vocal amplitude likewise stress that accurate interpretation of song amplitude depends on careful consideration of recording geometry, spectral composition and analytical choices, including the choice of weighting scale (Zollinger and Brumm 2015; Brumm et al. 2025). Our findings therefore reinforce the importance of explicitly reporting weighting scales and aligning SPL measurements with the perceptual and ecological context of the study species, especially when amplitude is treated as a biologically informative trait.

Although these differences follow from the filter characteristics, they inform the choice of scale. Z‐weighted levels were consistently the highest, and their maxima (95–116 dB at 1 m, compared with 87–95 dB for A‐weighted values; Table 1) indicate that unweighted measurements are inflated by low‐frequency energy, such as wind and distant anthropogenic noise, that is unrelated to the song. C‐weighted levels were consistently 0.7–1.4 dB below A‐weighted levels, as expected from the slight gain of the A‐filter between c. 1 and 5 kHz, where most of the energy of the songs studied here is concentrated (Table 1). The A‐filter is approximately flat over this range and attenuates lower and higher frequencies (Brumm 2004), broadly matching the avian audiogram, in which sensitivity is greatest at c. 1–5 kHz and declines steeply at lower frequencies (Dooling et al. 2000). We therefore consider A‐weighting the most appropriate scale for measuring the amplitude of passerine song. It approximates the frequency range relevant to avian receivers, reduces the contribution of low‐frequency noise and ensures comparability with most previous studies (Brumm 2004; Brumm et al. 2025; Menezes and Podos 2026). C‐ or Z‐weighting may be preferable only for vocalisations with substantial energy below c. 1 kHz, such as those of many non‐passerines, and the weighting scale used should always be reported.

Finally, because our study was observational, the associations reported here are correlational. Several of the drivers identified could be disentangled experimentally. Noise playback with controlled spectral overlap and duration could test the Lombard response under natural conditions, while simulated territorial intrusions could manipulate social context, as in previous experiments (Brumm and Todt 2002; Brumm and Ritschard 2011; Acorn et al. 2026). Finally, repeated measurements of the same males across mornings could separate effects of time of day from those of temperature and humidity.

## 5 Conclusions

Taken together, these results indicate that song amplitude is best understood as a flexible component of vocal performance shaped primarily by song structure and signalling context, rather than as a fixed or purely allometric trait. Temporal and spectral properties of song emerged as the strongest predictors of amplitude, whereas environmental effects were strongly species‐specific. Body size accounted for interspecific variation only when the Great Reed Warbler was included, while within‐species analyses revealed substantial scope for short‐term amplitude modulation, consistent with behavioural regulation rather than strict morphological constraint.

Differences among weighting scales, although expected from the filter characteristics, mean that amplitude values obtained with different scales are not directly comparable. Because the A‐filter broadly approximates avian hearing and reduces the contribution of low‐frequency noise unrelated to the signal, we recommend A‐weighted measurements for studies of passerine song amplitude, and explicit reporting of the weighting scale in all studies.

## Author Contributions

**K. Deoniziak:** conceptualization (lead), data curation (lead), formal analysis (equal), funding acquisition (lead), investigation (equal), methodology (lead), project administration (lead), supervision (lead), validation (lead), visualization (equal), writing – original draft (equal), writing – review and editing (equal). **W. Mierzejewska:** investigation (equal), writing – review and editing (equal). **M. Nartowicz:** investigation (equal), writing – review and editing (equal). **D. Winiarska:** formal analysis (equal), visualization (equal), writing – review and editing (equal).

## Funding

This research was funded by the National Science Centre, Poland, under project number 2022/47/D/NZ8/00565.

## Ethics Statement

This study was entirely observational and involved no capture, handling or manipulation of animals. According to Polish legislation, non‐invasive behavioural and acoustic observations of wild birds do not require ethical approval or special research permits. Fieldwork was conducted exclusively on publicly accessible land and on areas outside strictly protected zones with limited access. All procedures complied fully with national regulations governing wildlife research and animal welfare.

## Conflicts of Interest

The authors declare no conflicts of interest.

## Supporting information


**Table S1:** Summary statistics for ambient noise levels (dB(A)) measured within male territories for each study species. Ambient noise was measured as the maximum A‐weighted SPL (FAST, 125 ms) during the 1 s immediately after each recorded song, at the recording position.
**Table S2:** Summary of species‐level body measurements and corresponding PC1 scores. Values represent species means derived from the AVONET database. PC1 explained 74.9% of the variance (loadings: body mass 0.51, tarsus length 0.43, wing length 0.52, culmen length 0.54).
**Table S3:** Dunn post hoc tests with Holm correction comparing song amplitude at 1 m among weighting scales (A, C, Z) within species following significant Kruskal–Wallis tests.
**Table S4:** Summary of model 1 excluding the Great Reed Warbler.

## Data Availability

The dataset of song amplitude measurements, together with the associated environmental, contextual and acoustic variables, and the R code used for all across‐species and within‐species analyses are openly available in Zenodo at https://doi.org/10.5281/zenodo.23209667.
