# Supplementary material for: Ecological and Structural Drivers of Song Amplitude Variation in Passerines
Source: Ecol Evol. 2026 Nov 10;16(11):e74693. doi: 10.1002/ece3.74693 (PMC13749070; doi:10.1002/ece3.74693)
Supplement: Supplementary file 1 [file ECE3-16-e74693-s001.docx]

**Table S1.** Summary statistics for ambient noise levels (dB(A)) measured within male territories for each study species. Ambient noise was measured as the maximum A-weighted SPL (FAST, 125 ms) during the 1 s immediately after each recorded song, at the recording position.

| **Species** | **Scientific name** | **Mean** | **Min** | **Max** |
| --- | --- | --- | --- | --- |
| Common Chaffinch | *Fringilla coelebs* | 37.1 | 25.6 | 50.8 |
| Yellowhammer | *Emberiza citrinella* | 36.9 | 26.4 | 46.9 |
| Common Chiffchaff | *Phylloscopus collybita* | 41.5 | 30.5 | 55.8 |
| Willow Warbler | *Phylloscopus trochilus* | 38.0 | 24.2 | 55.1 |
| Common Whitethroat | *Curruca communis* | 40.3 | 28.4 | 58.8 |
| Great Reed Warbler | *Acrocephalus arundinaceus* | 42.2 | 29.9 | 59.5 |

**Table S2.** Summary of species‑level body measurements and corresponding PC1 scores. Values represent species means derived from the AVONET database. PC1 explained 74.9% of the variance (loadings: body mass 0.51, tarsus length 0.43, wing length 0.52, culmen length 0.54).

| **Species** | **Scientific name** | **Body mass (g)** | **Tarsus length (mm)** | **Wing length (mm)** | **Culmen length (mm)** | **PC1** |
| --- | --- | --- | --- | --- | --- | --- |
| Common Chaffinch | *Fringilla coelebs* | 23.8 | 17.8 | 85.9 | 14.7 | 0.214 |
| Yellowhammer | *Emberiza citrinella* | 29.7 | 19.1 | 88.2 | 13.4 | 0.581 |
| Common Chiffchaff | *Phylloscopus collybita* | 8.3 | 19.7 | 58.8 | 11.6 | -1.857 |
| Willow Warbler | *Phylloscopus trochilus* | 8.7 | 19.3 | 65.9 | 12.8 | -1.435 |
| Common Whitethroat | *Curruca communis* | 15.1 | 21.3 | 72.6 | 13.6 | -0.477 |
| Great Reed Warbler | *Acrocephalus arundinaceus* | 30.0 | 27.3 | 91.1 | 21.9 | 2.975 |

**Table S3.** Dunn post‑hoc tests with Holm correction comparing song amplitude at 1 m among weighting scales (A, C, Z) within species following significant Kruskal–Wallis tests.

| **Species** | **Weighting 1** | **Weighting 2** | **N** | **Z** | **P** |
| --- | --- | --- | --- | --- | --- |
| Chaffinch | A | C | 406 | -8.01 | <0.001 |
| Chaffinch | A | Z | 406 | 1.10 | 0.271 |
| Chaffinch | C | Z | 406 | 9.11 | <0.001 |
| Yellowhammer | A | C | 579 | -4.38 | <0.001 |
| Yellowhammer | A | Z | 579 | 12.84 | <0.001 |
| Yellowhammer | C | Z | 579 | 17.21 | <0.001 |
| Great Reed Warbler | A | C | 636 | -8.33 | <0.001 |
| Great Reed Warbler | A | Z | 636 | -0.36 | 0.715 |
| Great Reed Warbler | C | Z | 636 | 7.96 | <0.001 |
| Whitethroat | A | C | 823 | -8.37 | <0.001 |
| Whitethroat | A | Z | 823 | 5.57 | <0.001 |
| Whitethroat | C | Z | 823 | 13.94 | <0.001 |
| Chiffchaff | A | C | 357 | -6.59 | <0.001 |
| Chiffchaff | A | Z | 357 | 7.69 | <0.001 |
| Chiffchaff | C | Z | 357 | 14.28 | <0.001 |
| Willow Warbler | A | C | 464 | -8.69 | <0.001 |
| Willow Warbler | A | Z | 464 | 5.58 | <0.001 |
| Willow Warbler | C | Z | 464 | 14.27 | <0.001 |

**Supplementary Analysis: Model 1 – body size (PC1) excluding the Great Reed Warbler**

To examine whether the across‑species effect of body size on song amplitude was driven by any single species, we performed an additional analysis in which the Great Reed Warbler (*Acrocephalus arundinaceus*), the largest and loudest species, was removed from the dataset. We refitted the same Gaussian linear mixed model (LMM) using the standardised body‑size index and recordist identity as fixed effects, and species and male identity as random intercepts, following the same Bayesian framework described for the main analysis. The reduced dataset included 2629 amplitude measurements from five species and 100 individuals. The body-size index was re-standardised on the reduced data set. This model produced 175 divergent transitions (0.9% of post-warm-up draws), reflecting a between-species variance close to zero, and its estimates should therefore be interpreted with caution.

In contrast to the full across‑species model, body size no longer predicted variation in song amplitude when the Great Reed Warbler was excluded (Supplementary Table S4). The random‑effects structure indicated a substantial decrease in species‑level variance relative to the full model, suggesting that interspecific differences in amplitude were largely attributable to the inclusion of this species. Taken together, these results show that the positive relationship between body size and vocal amplitude observed in the main analysis was primarily driven by the Great Reed Warbler.

**Table S4.** Summary of model 1 excluding the Great Reed Warbler.

| **Model** | **Predictor** | **Posterior mean (95% CI)** |
| --- | --- | --- |
| Model 1 – body size | **(intercept)** | **81.45 (80.65 to 82.29)** |
|  | Body size (PC1) | -0.03 (-0.73 to 0.67) |
|  | Recordist | 0.09 (-0.88 to 1.02) |
